# Intellectual Development in Autism Spectrum Disorders: New Insights from Longitudinal Studies

**DOI:** 10.3389/fnhum.2013.00354

**Published:** 2013-07-05

**Authors:** Giacomo Vivanti, Josephine Barbaro, Kristelle Hudry, Cheryl Dissanayake, Margot Prior

**Affiliations:** ^1^Olga Tennison Autism Research Centre, School of Psychological Science, La Trobe University, Melbourne, VIC, Australia; ^2^Victorian Autism Specific Early Learning and Care Centre, La Trobe University, Melbourne, VIC, Australia; ^3^Melbourne School of Psychological Sciences, University of Melbourne, Melbourne, VIC, Australia

**Keywords:** autism, intellectual disability, cognitive development, comorbidity, developmental cognitive neuroscience

## Abstract

The presence/absence of Intellectual Disability (ID) is considered to be the most critical factor affecting outcomes in individuals with Autism Spectrum Disorders (ASD). However, the question of the specific nature of ID in ASD has received little attention, with the current view being that ID is a comorbid condition (i.e., one that is unrelated in etiology and causality from the ASD itself). Recent advances in developmental neuroscience, highlighting the importance of early exposure to social experiences for cognitive development, support an alternative view; that ID in ASD might emerge as a consequence of severe social-communication deficits on the experience-dependent mechanisms underlying neurocognitive development. We tested this prediction in two independent samples of young children with ASD (*N*s = 23 and 60), finding that children with greater ASD severity at an initial assessment were more likely to present with poorer cognitive outcomes at a later assessment, irrespective of initial cognitive level. The results of this proof of principle study suggest that ASD symptom severity contributes to the extent to which the environmental input required to support “typical” brain development can be processed by the individual, so that the risk of developing ID increases as the number and severity of ASD social-communicative impairments increase.

## Introduction

Intellectual Disability (ID) is characterized by significant limitations in intellectual functioning and in adaptive behavior as expressed in conceptual, social, and practical adaptive skills, with age of onset before age 18 years (Schalock et al., 2010). Approximately two thirds of individuals with Autism Spectrum Disorders (ASD) have co-occurring ID (Dykens and Lense, 2011), and the presence/absence of ID is considered to be the most critical factor affecting outcomes in this population (Howlin et al., 2004; Henninger and Taylor, 2013). However, the question of the nature of the association between ID and ASD has received little attention. One common view in the current conceptualization of ASD is that ID is a *comorbid* condition that occurs over and above ASD symptomatology in some individuals with ASD (Nordin and Gillberg, 1998; Cashin et al., 2009; Matson and Worley, 2013). The term “comorbidity” is used in medicine to denote clinical entities “unrelated in etiology or causality to the principal diagnosis” (e.g., cancer diagnosed after a stroke), and therefore conceptually distinct from complications or sequelae of the principal diagnosis (Greenfield, 1989; Iezzoni, 1994, p. 52; see also Lilienfeld et al., 1994). Other authors suggest that ID and ASD are related in terms of their etiology (i.e., that which causes ID also causes ASD) but they are not themselves causally related (e.g., Waterhouse, 2013). The perspective according to which ID is a distinct additional entity to ASD is reflected in many aspects of ASD research. For example, many studies report that participants with “comorbid ID” were excluded, to allow for the study of “pure autism”; that is, autism not confounded by ID.

The main argument supporting the idea of ID as a comorbid feature of ASD is the notion that a person can have either one without also having the other. While we do not disagree that such dissociation is possible, we argue that the presence of such a situation is not sufficient to demonstrate the independence of the two conditions. We will provide a number of counter arguments supporting an alternative view that severe ASD symptoms increase the risk of an individual also developing ID. We propose a theoretical model indicating the specific mechanisms through which the risk of having ID is related to the severity of ASD symptoms, and we provide novel data from two independent studies to support this model.

### Theoretical arguments for the association of ID and ASD

The notion of ID and ASD as independent clinical entities reflects a modular conceptualization of cognition, according to which one processing domain/module (in the case of ASD, the processing of social information) can be selectively disrupted without negative repercussions on the rest of cognitive system (that is, other domains will not be affected). According to this framework, “pure autism” is the exemplification of a “modular impairment” involving selective difficulties with social processing, while in the situation of “autism confounded by ID,” additional (non-social) processing domains happen to be disrupted as well, albeit for a different reason (i.e., the occurrence of a distinct clinical entity which is causally unrelated to ASD). However, an alternative to this account on the relationship between ID and ASD can be advanced within a developmental neuroscience framework (see Thomas and Karmiloff-Smith, 2002). Recent research emphasizes the experience-dependent nature of early brain development (e.g., Grossmann and Johnson, 2007; Kuhl, 2007; Makinodan et al., 2012), pointing to the crucial role of early exposure to social experience opportunities for cognitive development. For example, education and active engagement in a socially rich environment is associated with both structural and functional brain changes, whilst rearing in minimally stimulating environments (e.g., some orphanages) has a negative impact on brain functioning (Blakemore and Frith, 2005; Cicchetti and Cohen, 2006; Nelson, 2007).

Given the relevance of social input for normal brain development (Kuhl, 2007), it has been hypothesized that a decrease in the attentional and processing weight assigned to social information, in children with ASD, might preclude the usual social experiences that are necessary for “normal” cognitive development during early sensitive periods (Dawson, 2008; Klin et al., 2009; see also Hobson, 2004). This process would affect a number of different domains. For example, as noted by Whitehouse et al. (2007) language impairments in this population might be a secondary consequence of ASD symptoms. If infants with ASD do not have access to the appropriate input that supports the efficient organization and specialization of the brain in neurotypical development, this might ultimately result in the child also having an ID. A corollary of this model is that the more severe the ASD symptoms, the more the child would be “at risk” for developing an ID. Therefore, according to this view, ID is not a comorbid condition (i.e., an unrelated clinical entity), but a developmental *consequence* of the virtual “social deprivation” caused by the ASD symptoms. In this model, we do not imply that the presence of ASD necessarily results in ID in all children, regardless of symptom severity. Rather, we argue that ASD symptoms put the child “at risk” for ID, and that this risk will increase as the severity of ASD symptoms increase. One objection to this perspective is that the severity of ASD and ID are unrelated, so that ID can be equally present in children with mild or severe ASD symptoms. We turn to recent literature suggesting that this is not the case.

### Empirical arguments for the association of ID and ASD

If the notion that ID is a comorbid feature of ASD is correct, then measures of ASD severity and of cognitive abilities should be independent in the ASD population, so that a child could have mild ASD with severe ID, or severe ASD with mild ID. Empirical data, however, appear more consistent with our reasoning, showing that ID is more likely to be present in children with more severe ASD symptoms than in those with milder presentations. A review by Dykens and Lense (2011) using diagnostic categories from DSM-IV-TR (American Psychiatric Association, 2000) indicates that IQ levels vary substantially across diagnoses under the umbrella of the Pervasive Developmental Disorders (i.e., ASD), with more severe forms associated with lower cognitive scores. Furthermore, a recent longitudinal study by Gotham et al. (2012), involving a sample of 345 participants, documented that individuals with more severe autism symptoms had lower IQ, leading the authors to conclude that autism characteristics and cognitive functioning are not entirely independent features.

Another argument supporting our position derives from recent research on intervention in this population by Dawson et al. (2010). This study, focusing on the efficacy of the Early Start Denver Model – an intervention program specifically targeting ASD symptoms in very young children – found that children undergoing this program experience significant gains on measures of cognitive development and adaptive behavior. If ASD symptoms and ID (defined by low cognitive ability and adaptive functioning) are independent features, how is it that intervention targeting ASD symptoms results in gains in cognitive and adaptive functioning?

### Current aims and hypotheses

Based on the aforementioned arguments, we conducted a proof of principle study testing the hypothesis that severity of ASD in early childhood is associated with poorer development of cognitive abilities. We did this by collating secondary data across two independent samples of young children with ASD, each followed longitudinally. We predicted that (1) children with more severe ASD symptom presentation would be more likely to also demonstrate lower overall levels of cognitive ability, and (2) that these children would make slower gains in cognitive skills across time.

## Materials and Methods

### Participants

Data for this study were available from two pre-existing, independent samples of young children with ASD; one comprising preschoolers with ASD diagnoses given by expert autism assessment teams in the community, and the other comprising toddlers with ASD prospectively identified and diagnosed from a low-risk, community-based sample following developmental surveillance. Ethics approval for each of these was provided via the La Trobe University Human Ethics Committee; HEC 10-084 and HEC06-94, respectively. We present data from each study as collected across two visits (hereafter, Time 1 and Time 2). Initial characterization data for each sample at Time 1 is presented in Table 1.

**Table 1:** **Sample characteristics at time 1; mean (SD) range**.

	Preschoolers with ASD (community diagnosis)	Toddlers with ASD (developmental surveillance)
Chronological age (months)	40 (11) 22–60	25 (2) 23–33
ADOS-G^a^ total algorithm	14.9 (4.6) 6–21	14.0 (4.1) 6–20
MSEL^b^ Age-equivalence (months)		
Overall mental age	21.4 (12) 8–54.5	16.7 (3.4) 8–26
Subscales		
Visual reception	22.6 (8.9) 10–54	18.9 (3.2) 10–29
Fine motor	26.3 (10.7) 13–68	21.6 (3.7) 9–30
Receptive language	17.4 (10.7) 1–47	12.2 (5.1) 1–25
Expressive language	19.8 (11.5) 3–43	14.2 (4.1) 3–26

*^a^Autism diagnostic observation schedule – generic (Lord et al., 1999)*.

*^b^Mullen scales of early learning (Mullen, 1995)*.

#### Sample 1: preschoolers with community-based ASD diagnosis

Data for 23 children with ASD (1 female), aged 22–60 months at Time 1, were available from their enrollment and ongoing participation at a community early intervention center (see Vivanti et al., 2013). Children were accepted into the center on the basis of having an existing (or provisional) ASD diagnosis given by a community professional. This was confirmed at Time 1 via administration of the Autism Diagnostic Observation Schedule – Generic (ADOS-G; Lord et al., 1999) by an independent clinician with demonstrated research-reliability in the use of this measure. All participants were free from other medical conditions, and any visual, hearing, or motor impairment. Cognitive abilities for each child were assessed at entry to the early intervention center (Time 1) and again 1 year later (Time 2).

#### Sample 2: toddlers with ASD identified via developmental surveillance

Data for 60 toddlers with ASD (15 female), aged 23–33 months at Time 1, were drawn from a larger pool of participants identified within the Social Attention and Communication Study (SACS; Barbaro and Dissanayake, 2010; Barbaro et al., 2011). This longitudinal, community-based developmental surveillance study investigated the utility of a set of early markers in infants and toddlers for the prospectively identification of ASD at 12-, 18-, and 24 months of age. A sample of 110 children were identified at “at risk” for ASD on the SACS, and assessed at 24 months using the ADOS-G (Lord et al., 1999) and ADI-R (Lord et al., 1994), administered by a researcher with demonstrated reliability in using these measures. Of these, 89 toddlers were identified as having ASD at 24 months of age (Time 1), with 60 returning for a follow-up assessment 2 years later (Time 2; mean age 50 months, SD = 4.8). The current sample therefore comprises those 60 toddlers for whom an ASD diagnosis was initially given and for whom longitudinal cognitive ability data were available at follow-up.

### Measures

Common measures of autism symptoms and cognitive ability were used to characterize children across the two samples. As already noted, the ADOS-G (Lord et al., 1999) was administered at Time 1 for all children (early intervention entry for the preschoolers, and initial diagnostic assessment visit for the toddlers). This standardized tool is considered the gold-standard observational measure for use in quantifying symptoms relevant to a diagnosis of ASD; that is, impairments in the areas of communication, reciprocal social interaction, and restricted/repetitive behaviors. While serving to inform diagnostic decisions around ASD, ADOS-G total algorithm scores (comprising communication and social interaction items only) can also be considered to index relative symptom severity, in so far as a range of scores is available beyond that considered to signal the “cut-off” for an ASD. Total algorithm scores for Module 1 (minimally verbal young children) plausibly range from 0 to 26, with a cut-off score of 7 used to identify an ASD (and with higher scores indexing greater symptom expression). As shown in Table 1, Time 1 ADOS scores varied substantially across the available range within each of our samples, showing clear individual differences on this measure.

Cognitive ability was assessed for all children at both Time 1 and Time 2 (following 1 year of early intervention for the preschoolers, and at a 2-year follow-up, post diagnosis, for the toddlers). This was achieved using the Mullen Scales of Early Learning (MSEL; Mullen, 1995), a standardized measure of ability across four domains important for cognitive functioning in early development; Visual Reception, Fine Motor, Receptive Language, and Expressive Language. The MSEL yields standardized *T*-Scores for each domain. However, as these can be of limited use for samples where children have ASD, as floor-level performance is often observed, we instead report Age-Equivalence (AE) scores here. These scores demonstrate good variability and can be meaningfully interpreted (i.e., a typically developing child should be expected to have an AE score in line with their own chronological age, and to make gains of 12 months’ AE over a 1-year period). As shown in Table 1, Time 1 MSEL scores varied substantially, showing clear individual differences on this measure within each sample.

### Design and analytic procedure

Associations between autism symptom presentation and cognitive ability were evaluated, separately within each sample, using Pearson Product Moment Correlation Coefficients. First, we examined concurrent associations among our indices of autism symptoms and cognitive ability at Time 1. Second, we computed a measure of gains in cognitive ability between Time 1 and 2 assessments (subtracting the former from the latter, for each child) and then examined the association of ASD symptoms at Time 1 with this measure of cognitive AE gains.

## Results and Discussion

### Sample 1: Preschoolers with community-based ASD diagnosis

**Figure 1:**
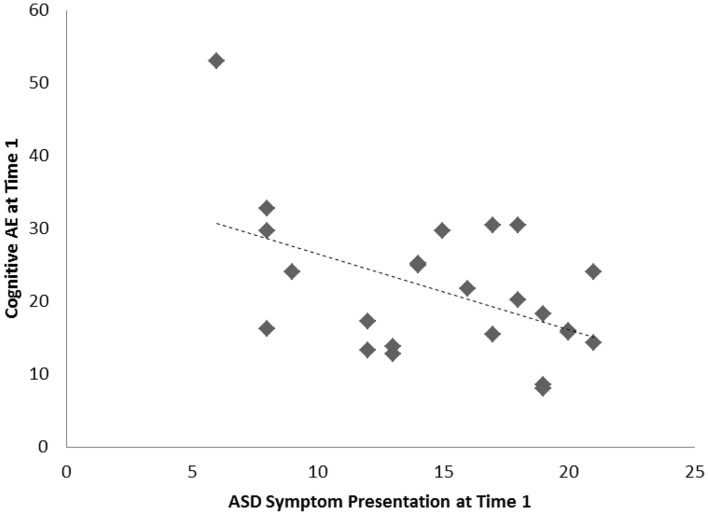
**Scatterplots for 23 preschoolers with community ASD diagnoses presenting associations among Time 1 ASD symptoms and concurrent cognitive age-equivalence scores**.

**Figure 2:**
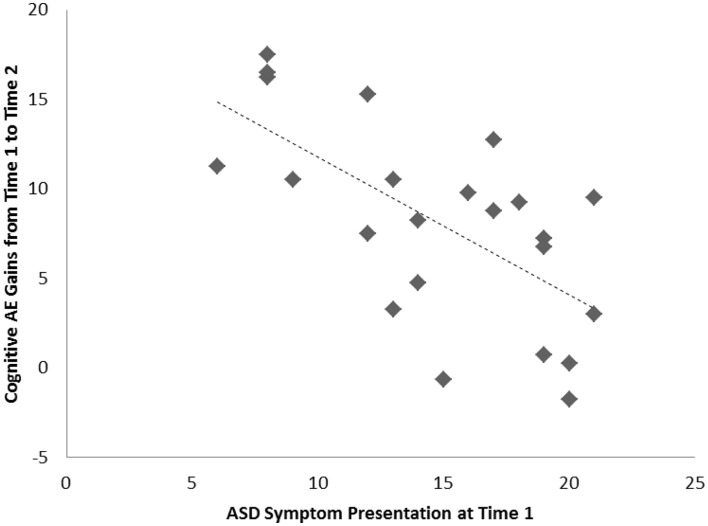
**Scatterplots for 23 preschoolers with community ASD diagnoses presenting associations among Time 1 ASD symptoms and gains in cognitive age-equivalence over a 1-year follow-up period**.

For the sample of 23 preschoolers with ASD, a significant association, of large effect size, was evident between ASD symptoms and cognitive AE scores assessed concurrently at Time 1 (i.e., early intervention intake); *r* = −0.49, *p* = 0.010, *d* = 0.9. Longitudinally, a significant association, of larger effect size, was evident for this sample between Time 1 ASD symptoms and gains in cognitive AE made between Time 1 and Time 2 assessments; *r* = −0.65, *p* = 0.001, *d* = 1.2. Average cognitive AE scores increased from 21.4 months (SD = 12) to 30.4 months (SD = 18) over the 1-year period, representing an average growth of 9 months within this time. The results from the correlational analyses indicated that those preschoolers with more severe ASD symptoms also presented with lower concurrent cognitive ability. Moreover, they also had fewer gains in cognitive ability over the following year. Figures 1 and 2 present individual data-points for these associations.

### Sample 2: Toddlers with ASD identified via developmental surveillance

**Figure 3:**
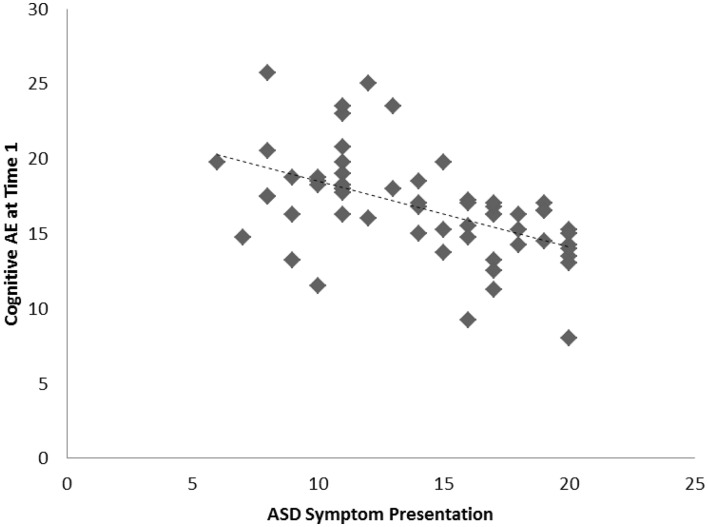
**Scatterplots for 60 toddlers with ASD identified via developmental surveillance presenting associations among Time 1 ASD symptoms and concurrent cognitive age-equivalence scores**.

**Figure 4:**
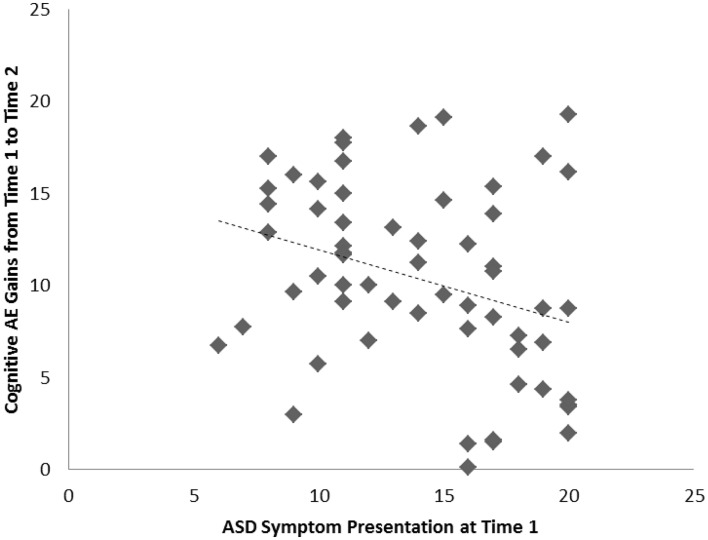
**Scatterplots for 60 toddlers with ASD identified via developmental surveillance presenting associations among Time 1 ASD symptoms and gains in cognitive age-equivalence over a 2-year follow-up period**.

Among the sample of 60 toddlers prospectively identified via developmental surveillance and meeting criteria for ASD at 2 years of age, a significant association, of medium effect size, was evident among concurrent ASD symptoms and cognitive AE assessed at Time 1 (i.e., initial diagnostic assessment); *r* = −0.52, *p* < 0.001, *d* = 0.7. A significant association, also of medium effect size, was also evident for this sample between ASD symptom presentation at Time 1 and gains in cognitive AE made between Time 1 and Time 2 assessments; *r* = −0.32, *p* = 0.007, *d* = 0.7. On average, cognitive AE scores increased from 16.7 months (SD = 3.4) to 37.5 months (SD = 11.5) over the 2-year period, representing an average growth of 20.8 months during this time (or around 10.4 months per year). As with our findings for the preschoolers with ASD, and in keeping with our hypothesis, toddlers with more severe ASD symptoms showed lower cognitive ability at concurrent assessment and also made more limited cognitive gains across the following 2 years. These associations are shown in Figures 3 and 4, and a summary of our data across both samples is presented in Table 2.

**Table 2:** **Summary of the strengths of association across the two samples**.

	Preschoolers with ASD (community diagnosis)	Toddlers with ASD (developmental surveillance)
Concurrent associations		
Time 1 ADOS^a^ and MSEL^b^	*r* = −0.49, *p* = 0.010, *d* = 0.9	*r* = −0.52, *p* < 0.001, *d* = 0.7
Longitudinal associations		
Time 1 ADOS^a^ and Time 2 MSEL^b^	*r* = −0.65, *p* = 0.001, *d* = 1.2	*r* = −0.32, *p* = 0.007, *d* = 0.7
Interval between Time 1 and 2	1 year	2 years

*^a^Autism diagnostic observation schedule – generic (Lord et al., 1999)*.

*^b^Mullen scales of early learning (Mullen, 1995)*.

### Cross-sample similarities and differences

Across these two independent samples of young children with ASD, similar magnitude of associations between Time 1 ASD symptom scores and concurrent cognitive AE are noted. However, effect sizes for the correlations between Time 1 ASD symptoms and longitudinal gains in cognitive AE were of quite different magnitude, albeit presenting a negative association for both groups. A somewhat stronger effect was observed here for our sample of preschoolers, followed across a 1-year period, with a more modest one apparent for our toddlers who were observed across a 2-year period. This variation in effect sizes may be due to sample differences. First, while our toddler sample included a greater number of individuals (*N* = 60) than our preschool sample (*N* = 23), the former comprised a relatively homogenous group, while the latter presented greater heterogeneity in terms of chronological age at each assessment and Time 1 cognitive AE scores (see standard deviation metrics in Table 1). Differences in the range of scores present within the groups may account, at least in part, for the differing magnitudes of effect size we observed. Variability in Time 1 ASD symptom presentation was relatively well balanced across the samples. However, by Time 2, cognitive AE scores were more varied in each of the groups. As such, this is unlikely to provide a full explanation for our finding.

The most likely potential contributing factor for the difference in effect size lies in the different intervals spanning Time 1 and Time 2 assessments for each group. In the case of the preschoolers, intake assessment to the community center was followed with an outcome evaluation 1 year later. For toddlers, by contrast, ASD diagnosis was made at a visit when children were ∼2 years of age (having been referred to the study across infancy and toddlerhood via developmental surveillance in the SACS) and the follow-up visit was scheduled ∼2 years later. This longer-interval, over which gains were evaluated, may explain the more modest effect size observed here between early ASD symptom presentation and change in cognitive AE ability.

## General Discussion

Autism Spectrum Disorder is defined in terms of limitations in social-communication and behavioral flexibility, and ID is defined in terms of limitations in intellectual functioning and in adaptive behavior as expressed in conceptual, social, and practical skills. Whilst the validity of ASD as a specific construct independent from ID and other factors (e.g., language) is well established, the majority of individuals with ASD also have co-occurring ID, posing the problem of the nature of this association. One possibility, which is widely accepted in the ASD field, is that ID represents a comorbid condition in ASD (i.e., one that is unrelated in etiology and causality from ASD). In this paper, we outlined a number of theoretical and empirical arguments indicating that this notion is questionable. Whilst there is clear evidence that mild ASD symptoms are compatible with normative or superior IQ, the evidence is less clear with regards to severe ASD symptoms, with recent data (including the novel data presented here) suggesting that the more “autism specific” symptoms a child has, the more at risk he or she is of poor cognitive outcome. Moreover, early behavioral intervention targeting ASD symptoms results in positive changes in IQ, again indicating some inter-dependence, rather than independence, of these two dimensions.

We argue that the presence of severe ASD symptoms is a risk factor for low IQ, in the same way that severe hypertension or severe obesity increases the risk of cardiovascular events (Flynn et al., 2011). Advances in developmental neuroscience, emphasizing the experience-dependent nature of early brain development (e.g., Grossmann and Johnson, 2007; Kuhl, 2007), allow us to explain this association of ID and ASD from a neurodevelopmental perspective. Based on this framework, we suggest that ASD symptom severity moderates the extent to which the environmental input required to support “typical” brain development can be processed by the individual, so that the risk of poor cognitive developmental outcomes increases as the number and severity of ASD social-communication impairments increase. That is, emerging social-communication deficits early in development might deprive the developing brain from receiving important environmental inputs, with downstream effects on global cognitive development. The result is lower IQ in those individuals with the most severe ASD symptoms.

According to this perspective, we argue that the practice of excluding children with ID in ASD research to study “pure autism unconfounded by ID” is ill considered, just as studying the risk of cardiovascular events in individuals who are slightly overweight, or who have mild presentation of hypertension, would not be informative on the most relevant aspects affecting the outcomes of individuals with those conditions. Rather, research should target those factors that place affected individuals at an increased risk of negative outcomes, by investigating the mechanisms underlying symptoms and their sequelae, and identifying prevention/remediation strategies to foster positive developmental outcomes.

### Limitations and future directions

There were several limitations of the current study. First, we do not have data on participants’ adaptive behavior level, which form part of the criteria required for the diagnosis of ID. Second, whilst all of the children involved in this study would likely have been enrolled in community early intervention programs, we have not collected details on the specific programs the children were involved in, nor on the amounts of intervention being received. As such, this potentially critical factor mediating the impact of ASD severity on cognition could not be taken into account in the current study. Future research will involve collecting data on adaptive behavior and participation in intervention in order to build on the results from the current study.

Whilst the aim of this proof of principle study was to test the notion that the severity of ASD symptoms has a negative influence on cognitive development, it is important to mention various other risk factors that are known to affect cognitive development in other groups of children. These include demographic factors, preterm birth, maternal age and education, and birth weight, among others (Zeanah, 2009). In order to disentangle the relative importance of ASD symptom severity from that of other potentially significant factors on cognitive development, more empirical research is needed that utilizes prospective research designs and includes larger sample sizes.

## Conclusion

Intellectual Disability is known to result from a number of different risk factors. Here, we have argued that the presence of severe (but not mild) symptoms of ASD is one such risk factor, so that ID is unlikely to be a comorbid condition to ASD but, rather, one that is intimately linked to certain ASD presentations. Consistent with Ockham’s razor (Popper, 1992), the presence of ID in the majority of the ASD population, in particular in those individuals who are more severely affected with ASD, can be more parsimoniously explained by positing a relationship between these two frequently co-occurring clinical entities than by claiming their independence. A developmental neuroscience framework provides a good explanatory model on the nature of such a relationship, indicating that it is plausible that children with very severe disabilities affecting social understanding and social learning are more vulnerable to poor cognitive outcomes (Coch et al., 2007). As the poor outcomes associated with the presence of ID in ASD result in large human and societal costs, it is important that future research systematically investigate the risk and protective factors associated with the development of ID in ASD. Indeed, excluding individuals with ID from research in ASD only renders more difficult the ultimate goal of fostering positive outcomes for individuals with ASD.

## Conflict of Interest Statement

The authors declare that the research was conducted in the absence of any commercial or financial relationships that could be construed as a potential conflict of interest.
